# A Role of Lipid Metabolism during Cumulus-Oocyte Complex Maturation: Impact of Lipid Modulators to Improve Embryo Production

**DOI:** 10.1155/2014/692067

**Published:** 2014-03-06

**Authors:** E. G. Prates, J. T. Nunes, R. M. Pereira

**Affiliations:** ^1^INIAV Instituto Nacional de Investigação Agrária e Veterinária, Unidade de Biotecnologias e Recursos Genéticos-Santarém, Quinta da Fonte Boa, 2000-048 Vale de Santarém, Portugal; ^2^Instituto de Ciências Agrárias e Ambientais Mediterrânicas (ICAAM), Universidade de Évora, Núcleo da Mitra, Ap. 94, 7002-554 Évora, Portugal; ^3^Escola Universitária Vasco da Gama, Mosteiro de S. Jorge de Milréu, 3040-714 Coimbra, Portugal

## Abstract

Oocyte intracellular lipids are mainly stored in lipid droplets (LD) providing energy for proper growth and development. Lipids are also important signalling molecules involved in the regulatory mechanisms of maturation and hence in oocyte competence acquisition. Recent studies show that LD are highly dynamic organelles. They change their shape, volume, and location within the ooplasm as well as their interaction with other organelles during the maturation process. The droplets high lipid content has been correlated with impaired oocyte developmental competence and low cryosurvival. Yet the underlying mechanisms are not fully understood. In particular, the lipid-rich pig oocyte might be an excellent model to understand the role of lipids and fatty acid metabolism during the mammalian oocyte maturation and their implications on subsequent monospermic fertilization and preimplantation embryo development. The possibility of using chemical molecules to modulate the lipid content of oocytes and embryos to improve cryopreservation as well as its biological effects during development is here described. Furthermore, these principles of lipid content modulation may be applied not only to germ cells and embryo cryopreservation in livestock production but also to biomedical fundamental research.

## 1. Introduction

Oocyte quality is one of the key limiting factors in female fertility [1, 2]. The ovarian follicular microenvironment and maternal signals, mediated primarily through granulosa and cumulus cells (CC), are responsible for nurturing oocyte growth and its gradual acquisition of developmental competence [1].* In vitro* maturation (IVM) of oocytes can provide large numbers of mature oocytes which are capable of supporting embryo development and full development to term [3]. In livestock production, these techniques can be useful in breeding programmes and animal genetic cryoconservation [4]. However, the high intracellular lipid content of oocytes and embryos has been reported to impair cryopreservation, with particular relevance in pig [5, 6]. Different strategies can be used to manipulate oocyte or embryo lipid contents. Nevertheless, a role for lipids in energy production during preimplantation development as well as precursors in steroidogenic and eicosanoid pathways has been proposed [7–9], suggesting that modifications in oocyte intracellular lipids should be carefully estimated. The high intracellular lipid content of pig oocytes [10, 11] renders them an excellent model among mammalian and microlecithal oocytes to understand the role of lipids and fatty acid metabolism during maturation. Furthermore the oocyte activating* vias* seem to be closely related to those regulating the mobilization of intracellular lipid reserves in the maturating oocyte [12, 13]. This review will focus on the effects of lipid modulation by chemical molecules during oocyte culture in competence acquisition for monospermic fertilization and preimplantation embryo development. Various aspects of lipid droplets (LD) biogenesis and function in the cellular lipid metabolism during these processes will be further discussed in eligible species for assisted reproductive technologies (ART).

## 2. Oocyte Quality and Developmental Competence Acquisition

### 2.1. Morphological and Functional Characterization of Cumulus-Oocyte Complex

The cumulus-oocyte complex (COC) composed of the female gamete and the surrounding cumulus cells (CC) is a complete functional and dynamic unit playing a pivotal role in oocyte metabolism during maturation. The bidirectional exchanges of nutrients and regulatory molecules between oocyte and contiguous CC are crucial for oocyte competence acquisition, CC expansion, and early embryonic development [3, 14, 15]. In addition, the presence of CC during IVM was found to be effective in regulating the synthesis and concentration of important cytoplasmic factors such as glutathione (GSH) and Ca^2+^ [16]. Denuded mature oocytes unquestionably present differences in Ca^2+^ homeostasis. In fact, the duration of Ca^2+^ rise was reported to be higher but with lower amplitude in denuded mature pig oocytes compared with those matured in the presence of CC: COC or denuded oocytes cultured with CC added to culture medium [17]. Also, the activation of denuded mature oocytes mediated through Ca^2+^ peaks seems to be hampered, interfering with cytoskeleton and organelles migration, namely, LD and cortical granules, with repercussions in membrane block to polyspermy.

During* in vitro* culture of COC, CC underwent a molecular maturation process concomitantly with oocyte nuclear maturation. Additionally, oocytes actively regulate fundamental aspects of CC function via oocyte-secreted factors, controlling the COC microenvironment. In turn, the CC gene expression profile varies according to the stages of oocyte maturation [3, 15]. Ouandaogo et al. [15] used microarrays to identify a specific signature of 25 genes expression in CC issued from metaphase II (MII) oocytes compared with germinal vesicle and metaphase I. This CC expression profile can be useful as predictors of oocyte quality [3, 15]. Furthermore, the simultaneous expansion of compact layers of CC surrounding the oocyte and deposition of mucoelastic material in the extracellular matrix is implicated in supporting both the nuclear maturation and the cytoplasmic maturation [3, 17, 19]. The beneficial effect of CC during oocyte growth to stimulate competence acquisition to further support embryonic development is therefore unequivocal.

### 2.2. Oocyte Nuclear Maturation

Oocyte competence to complete nuclear maturation is acquired at least in two steps: firstly, oocytes are able to resume meiosis, undergo germinal vesicle breakdown (GVBD), and progress to metaphase I; secondly, oocytes are competent to advance beyond metaphase I, enter anaphase, and proceed to MII [20]. At the end of the maturation period, the meiotic spindle and chromosomal rearrangement at MII, as well as the first polar body, can be observed. Simultaneous with meiosis the cytoplasmic maturation proceeds. However, when oocytes are collected from ovaries and placed in culture they immediately reinitiate meiosis while cytoplasmic maturation is delayed [21].

Several mediator factors are involved in the maturation of an oocyte [22]. A critical signalling compound is the gonadotropin second messenger, cyclic AMP (cAMP), which is synthesized in the oocyte and in adjacent CC through the activation of the constitutively expressed transmembrane G-protein-coupled receptor [22–24]. The newly synthesized cAMP stimulates the cAMP dependent protein kinase A (PKA), whose type I mediates the inhibitory action on oocyte GVBD, while type II regulates the meiosis-inducing pulse of cAMP occurring within CC following hormonal stimulation [12]. Furthermore, the activity of AMP-phosphodiesterase (PDE) within oocytes hydrolyses cAMP to AMP, due to the epidermal growth factor (EGF) stimuli, and inactivates the PKA protein. A positive stimulus for oocyte nuclear maturation progression concomitantly with the GVBD is thus induced [12]. In addition, the AMP stimulates the mitogen-activated protein kinase (MAPK) pathway that can also be activated by growth factors or gonadotropin stimuli in CC [12, 25]. In the pig, MAPK sites of activation can be located either in the GV or in the cytoplasm, representing sites for nuclear and cytoplasmic maturation synchronization [12, 22, 25, 26]. Two isoforms of MAPK proteins were identified, the extracellular signal-regulated kinase ERK1 and 2 as being involved in the regulation of cell cycle and microtubule dynamics during metaphase organization [24, 26]. Furthermore, MAPK is implicated in retaining MII arrest, in the mature oocyte through the regulation of PDE action on cAMP degradation, and in the maintenance of maturation promoting factor (MPF) activity [12, 22].

### 2.3. Oocyte Cytoplasmic Maturation

The oocyte cytoplasmic maturation is a complex process comprising many organelles and compounds [2, 3, 17]. Asynchronous or incomplete cytoplasmic maturation has been indicated as a common phenomenon in the pig that can predispose oocytes to multiple sperm penetration through the zona pellucida into the cytoplasm before the block to polyspermy [21, 27, 28]. It is especially frequent under* in vitro* culture conditions [29, 30], as the prevalence of this pathological situation under natural conditions is moderate.* In vivo* polyspermy leads to the formation of polyploid embryos that die at a very early stage of development [27]. The physiological changes in oviductal fluid composition (namely, proteins, glycosaminoglycans, hormones, and growth factors) during oestrous play a critical role in gametes maturation and interaction to accomplish the monospermic fertilization. Modifications in oviductal fluid or fertilization media composition, spermatozoa concentration, interval between mating and fertilization that includes the period of spermatozoa capacitation, and the functional state of oocyte cortical granules can all account for polyspermy. Although extensive attempts have been made to reduce the penetration of pig oocytes by more than a single spermatozoon, the high incidence of polyspermy remains a major obstacle in* in vitro* embryo production at this species [21, 27, 30]. This problem is also present in aged female gametes of several species, including humans [2, 16, 31].

*In vivo*, oocytes reach their maturity within the antral stage of follicular development presenting different diameters according to species (mouse, hamster, cattle, sheep, pig, and human). The follicular environment influences oocyte growth and quality and thus its developmental capacity. The ooplasm of the growing follicle accumulates glycogen granules, LD composed of different FA, and proteins closely depend on the follicular fluid provision of metabolites [7, 14, 32, 33]. Full meiotic competence is reached in oocytes developing in ovarian follicles with a diameter of 3 mm or more [22, 25].* In vitro*, pig oocytes under 90 *μ*m in diameter are unable to resume meiosis, while oocytes measuring 110–115 *μ*m can complete the first meiotic division and acquire MII [10, 25]. However, many do not attain an optimal oocyte diameter before fertilization. During oocyte growth the enrichment in nutritive substances in the maturating cytoplasm is essential to support embryonic development. The cAMP level transiently delays the nuclear progression and synchronizes it with the synthesis in the cytoplasm [23, 34]. Furthermore, simultaneous with PKA stimulation, the protein kinase C (PKC) is activated, delaying meiotic progression and enhancing cytoplasmic maturation in pig and cattle oocytes [25, 35]. PKC is also involved in the regulation of cortical granules exocytosis during the oocyte fertilization process [36], and thus in the regulation of a monospermic penetration.

In spite of several efforts, oocyte cytoplasmic maturation remains a key limiting step for ART. The reasons why fully grown oocytes are not capable of becoming viable embryos are still elusive, but incomplete cytoplasmic maturation and/or asynchrony between nuclear and cytoplasmic maturation are certainly among those critically responsible.

### 2.4. Oocyte Quality Evaluation

**Figure 1:**
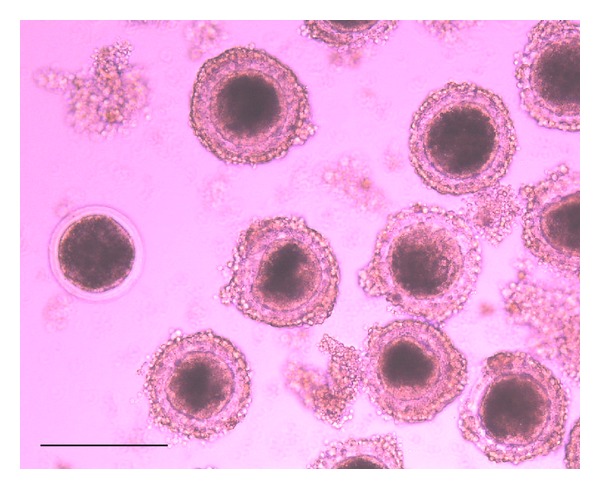
Morphological appearance of immature pig oocytes.

The use of morphological characteristics and metabolites involved in COC maturation can provide valuable information for the preselection of high-quality oocytes to maximize embryonic developmental outcomes [2]. Currently in* in vitro* embryo production techniques, COC are selected based on their morphological appearance (Figure 1). Different categories of COC can be distinguished, that is, good, fair, poor, and denuded by an expertise operator. During oocyte maturation, the cytoplasmic expansion can be measured through the diameter or area to predict oocyte competence or maturity. A relationship between oocyte area and its meiotic status has indeed been identified [32, 33].

Conversely, other morphological characteristics can be used to predict oocyte quality. The huge number of LD, as well as their distribution pattern or their interaction with other organelles and cytoplasmic pigments, is responsible for the dark colour tone that characterizes the oocyte of some mammalian species, namely, the pig, cattle, horse, or even of the minke whale [19, 37–39]. Although this colour tone seems to be species-specific, it has also been linked to oocyte quality. In fact, while a dark, almost black, granulated ooplasm is common in the pig oocyte due to its high lipid content, this is not the case in humans, where dark colour and cytoplasmic inclusions are related to low oocyte quality and fertility failure [2, 40]. Cattle and mare oocytes tend to present also a dark colour tone, although with different degrees of cytoplasmic transparency closely related to their maturation status and quality [19, 37, 39]. Even in pig, Cui et al. [17] showed that a bright gray and uniform ooplasm was a marker of better oocyte quality. Several techniques may be applied to compare lipid contents of oocytes from different donors, sized follicles, or cultured conditions. A lipid specific fluorescent dye, Nile red, was used to stain cattle, pig, and murine oocytes, and different amounts of emitted fluorescent light were measured according to their cytoplasmic lipid content [38]. In particular, the gray mean value within the oocyte fat area was suggested as an appropriate tool to evaluate the lipid content of a single oocyte [10]. Moreover, as this is a noninvasive technique, it can be useful to record oocyte morphology and quality before cryopreservation or fertilization. The possibility of subsequent use is of upmost importance in humans or endangered species due to the limited number of available oocytes.

Besides morphological characterization, metabolic markers are also eligible criteria to evaluate oocyte quality and estimate its fertilization ability [7]. Oocyte competence can be assessed by the brilliant cresyl blue test. This test relies on the measurement of a glucose-6-phosphate dehydrogenase (G6PDH) activity, an enzyme synthesized in the growing oocytes, but inactive in those that have finished their growth phase. The G6PDH converts the dye into a colourless form being the blue stained mature oocytes of higher quality [41, 42]. This test has been used in the pig to evaluate oocyte quality after modifications were made to maternal diet, and thus in the fatty acid (FA) profile of follicular fluid [42]. Interestingly, the FA profile in women changes in early midlife, before the age of 35, and these changes have been implicated in the decline of fertility [43]. Furthermore, the evaluation of other enzyme functions, such as Δ-9 and Δ-5 desaturase or its genetic expression monitorized by real-time PCR, as well as the lipid composition by gas chromatography analysis, can be used to complete the information about oocyte quality that seems to be closely related to its lipid composition [9, 11, 15, 42, 43].

## 3. The Role of Lipids during Oocyte Maturation and Initial Embryo Development

### 3.1. Oocyte and Embryo Lipid Metabolism

The pig oocyte is known as one of the most lipid rich oocytes in domestic animals [44]. The long preimplantation period observed in the pig, has been advanced as an explanation for this huge lipid content [39]. Moreover, due to the greater litter size, the huge lipid reservoir within the oocyte may be specifically required to provide energy until placenta development in the polytocous species, such as pig and dog. Eventually, embryo competition may occur for the implantation of the more competent embryos, thus resulting from the best oocytes. However, in oocytes from nonpolytocous species, like the horse [19] or the minke whale [37], the cytoplasm is also full of lipid inclusions, thus exhibiting a dark colour tone. The reasons for this great lipid content thus might be species specific, or even due to a phylogenic relation, as among the pig and horse ungulates.

**Figure 2:**
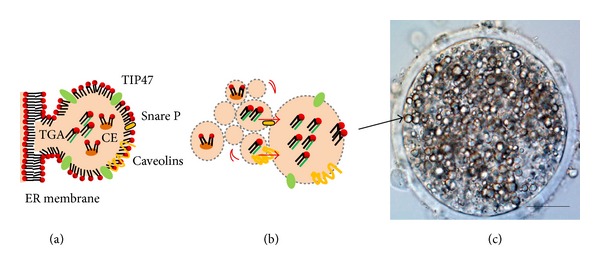
A model for lipid droplets (LD) biogenesis ((a), (b)) during pig oocyte (c) maturation: (a),* de novo* synthesis of LD in endoplasmic reticulum membrane and (b) LD coalescence process, with lipid trafficking mediated by snare (Snare P, orange batons) and caveolins (orange coiled lines) proteins (based on a study by Suzuki et al. [18]). Perilipins proteins (TIP47 green batons) involved in the mechanism of lipolysis regulation. (c) Pig oocyte with 24 hours of* in vitro* maturation. Scale bar 50 *μ*m. CE, cholesterol esters; ER, endoplasmic reticulum; TGA, triacylglycerols.

**Figure 3:**
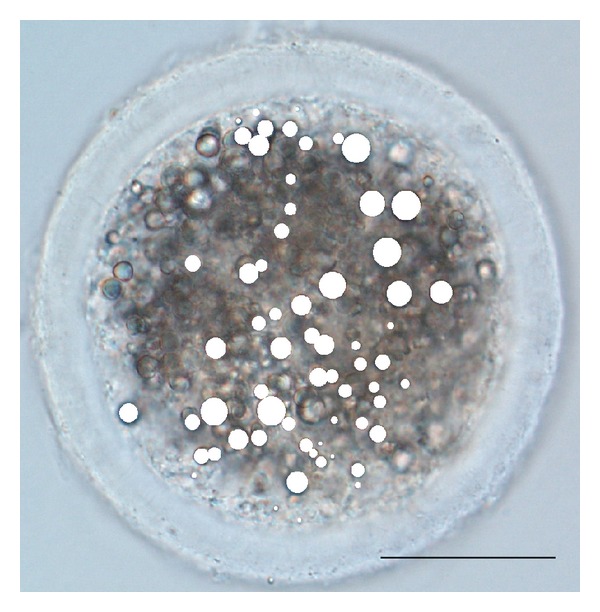
Immature pig oocyte with lipid droplets (LD) highlighted in white colour (LD areas were measured using Image J software) and scale bar 50 *μ*m.

Regarding LD composition, a core of neutral lipids is enveloped by a phospholipid monolayer containing a wide variety of proteins, embedded in both, the phospholipid monolayer and within the core [45]. The function of these cellular proteins in LD fractions is currently being studied. For instance, perilipins that are located at the LD surface in adipocytes and nonadipocytes were referred as having regulatory function in LD lipolysis [13]. According to this author, perilipin TIP47 was identified in steroidogenic tissues, and so a role for TIP47 in the oocyte lipolysis regulation may be expected during the maturation process. Moreover, LD are highly dynamic organelles. Indeed, LD can be* de novo* synthesized, refed with free FA, or grow through a coalescence process of existing droplets, mediated by SNARE proteins [46] (Figure 2). According to Suzuki et al. [18] LD are thought to be born in the endoplasmic reticulum (ER) membrane and it is likely that a protein based mechanism is involved in making lipid esters accumulate locally. Then, at an initial stage, lipid esters synthesized in the ER are deposited within its membrane. Afterwards they bud as a globule covered by the cytoplasmic phospholipids monolayer [18]. Regardless of LD origin, they are constantly changing their shape, volume, and location. In particular, in the lipid-rich pig oocyte the cytoplasm is filled with LD showing a considerable variation of areas, ranging from 0.3 *μ*m^2^ to 90 *μ*m^2^, during the maturation process [10] (Figure 3).

Lipid droplets can be found in association with other organelles linked to cellular metabolism such as mitochondria, ER, endosomes, peroxisomes, and cytoskeleton [19, 39, 45, 46]. During oocyte maturation, the activity and organization of LD and mitochondria are particularly relevant, since oxidative phosphorylation is the main pathway to supply ATP for cellular activities [2, 7]. In mature pig and mare oocytes two distribution patterns were identified for both LD and mitochondria: an even or homogenous distribution through the ooplasm and an uneven or heterogeneous allocation [17, 19]. The first distribution is more frequently observed in the pig [17]. Moreover, the evidence of regions of “colocalization” between LD and mitochondria and their relocalization during IVM was shown to be linked to intracellular oxygen gradients. Hence, Sturmey et al. [39] observed that the peripheral mitochondrial clustering in pig oocytes was correlated with higher oxygen availability in this region. The coexistence of LD and mitochondria in close proximity was also demonstrated in mature ruminant oocytes [47]. However, in the mare the majority of morphological normal IVM oocytes shows a polar aggregation of LD, localized independently of mitochondria that are placed in the hemisphere containing the meiotic spindle [19]. Further studies are needed to explain these discrepancies among species.

As referred, during human and pig oocyte aging, a dark colour tone became more pronounced and an irregular ooplasm was presented [2, 16]. On the other hand, the morphological changes observed in LD during oocyte maturation may reflect alterations in the nature of stored lipids [48]. The most abundant intracellular lipids stored within oocytes were shown to be the triacylglycerols, representing approximately 36 and 46% (w/w) of total FA in cattle and pig, respectively [44, 49]. These can be utilized in mitochondrial *β*-oxidation to produce energy during oocyte maturation [7, 39]. Furthermore, the enrichment in phospholipids and cholesterol during oocyte maturation is crucial to form membranes during the rapid cell divisions after oocyte fertilization [44]. Besides, phospholipids also play a role in the second messengers' synthesis during oocyte maturation and embryonic development. For instance, in pig oocytes, phosphatidylinositol represents 6% of total phospholipids, being rich in arachidonic (20:4 n-6) and stearic (18:0) acids and also in palmitic acid (16:0) [49]. Hydrolysis of this membrane phospholipid yields two second messengers, inositol 1,4,5-trisphosphate (IP3) and diacylglycerol (DAG). The IP3 and its derivative (IP4) increase Ca^2+^ concentrations, while DAG stimulates PKC [36, 44], these being particularly important in oocyte maturation and ability for monospermic fertilization.

Plasmalogens represent another important class of phospholipids in oocyte that has been identified as membranes constituents, both cytoplasmic and inner cell membranes, participating in the regulation of their dynamics [11, 49, 50]. They may release the ether vinylic (identified as dimethyl acetal by liquid-gas chromatography [11]), an analogous molecule to FA, from the membrane and accumulate in the citosol to form the diradylglyceride (DdGA). This compound competes with DAG for PKC site of activation, thus regulating the DAG and PKC activity [50]. Nevertheless, plasmalogens function during oocyte maturation and embryo development remains unknown. Additionally, membrane phospholipids composition might differ according to the oocyte maturation stage, immature and mature, but they are also dependent upon oocyte origin (species, breed, ovaries batch, and nutrition status) [11, 42, 44, 49].

Analysis of FA composition of cattle and pig oocytes during maturation showed that 16:0, followed by oleic (*c*9-18:1), 18:0 were the most abundant FA registered in immature and mature oocytes, followed by n-6 polyunsaturated fatty acids (PUFA), specifically linoleic (18:2 n-6) and 20:4 n-6, which may indicate that oocytes are capable of synthesizing prostaglandins (PG) and leukotrienes [9, 11, 44, 49]. Prostaglandins, namely, PGE_2_ can be critical mediators of CC expansion and of oocyte meiosis resumption and progression [8, 51]. Moreover, FA profile of cattle and pig oocytes were shown to change due to culture media or dietary lipid supplementation and differences in oocyte FA composition were related to oocyte developmental competence [9, 11, 42]. Although the role of lipids during oocyte maturation and early embryo development is currently under research, their primordial importance in female gamete quality is unequivocal. Modifications of LD morphology and lipid metabolism during pig oocyte maturation may interfere with monospermic fertilization, as referred, but also with embryo development [10, 11]. Immediately after sperm penetration, differences are identified in electron density of LD. The LD density is restored in the pronuclear stage, although the number and size of droplets seem reduced when compared to mature oocytes. At 2–4 cell and blastocyst stage, the features of LD are almost the same as those of pronuclear zygotes. However differences in LD content of blastomeres during early* in vitro* or* in vivo* embryonic development were reported [6, 31, 52].* In vitro*, the embryo culture media can also influence the size and number of LD, particularly by the provision of serum [6, 53]. Strong evidence suggests that preimplantation embryos are able to utilize FA stored endogenously or from the culture media as energy substrates [7, 53]. Notwithstanding differences in the metabolic pathways for energy production utilized during embryo development were identified. While during cleavage, the pentose phosphate pathway (PPP) is preferentially used; glycolysis and *β*-oxidation are prominent during subsequent embryo development. Their activity is intensified during compaction and blastocyst formation [7, 52, 54, 55]. This energy switch, occurring during the early embryo development, is believed to prepare the embryo for implantation, as well as to afford synthesis of macromolecules from glycolytic intermediates [56], and may be related/dependent upon the embryonic genome activation [31]. Adequate chemical or serum supplementation during culture is therefore crucial to allow proper embryo development [5, 52, 57].

### 3.2. Insight into Metabolic Disorders Related to Infertility

Infertility is a huge concern throughout the world both in animals and humans. Although the economic importance of the reproductive efficiency in livestock is recognised, herd fertility has declined over the past 30 years. Reduced oocyte and embryo quality were identified as major problems of this substandard fertility [9, 58]. Maternal metabolic disorders in high producing dairy cows, linked to the negative energy balance of postpartum or nutritionally induced, may alter the endocrine and biochemical composition of the follicular fluid, compromising both oocyte quality and embryo development [59].

Similar reports exist concerning the women fertility decline due to age and obesity [44, 60]. Furthermore the increasing incidence of premature signs of ovarian aging in younger women is also a matter of concern in terms of their reproductive performance [61]. The reason for such poor results is related to an aging population of oocytes of poor quality and a gradual depletion of the follicle pool. Age related changes in FA profiles, independently of the diet, as well as in key enzymes of lipid metabolism, were also identified [44, 61]. In several species obesity impairs both oocyte maturation and metabolism, negatively affecting further development [60]. Besides, both overweight and aged women present a higher percentage of oocytes with granular and dark cytoplasm showing reduced fertilizing ability either* in vivo* or* in vitro* [60, 61]. Aged oocytes from several species were also reported to present reduced capacity for the polyspermic blockage and altered lipid content [2, 16, 31]. Additionally the distribution and shape of mitochondria are also modified in these oocytes, changing from diffuse to aggregate and from spherical to elongated, respectively. The LD became solidified. These morphological alterations are concomitant to modifications in oocyte homeostasis, namely, in ATP synthesized from LD FA B-oxidation in mitochondria, in Ca^2+^ rise and amplitude regulating cytoskeleton and organelles migration, and thus in oocyte quality [16, 17].

Deregulation of neutral lipids storage in LD has been linked to a variety of metabolic diseases [62]. In fact, LD have been the focus of intensive research and it has become increasingly clear that the molecular machinery in and around LD regulates synthesis, utilization and trafficking of lipids and plays a crucial role in cellular lipid metabolism [18]. As previously mentioned oocyte quality and embryo development ability are undoubtedly related to LD dynamics and properties. Understanding the molecular mechanisms that regulate neutral lipids storage may hold the key to developing therapeutic tools for these metabolic disorders related to subfertility/infertility. In fact, new strategies to prevent and control infertility are urgently needed.

## 4. Lipid Modulators

### 4.1. Mechanisms of Action

**Figure 4:**
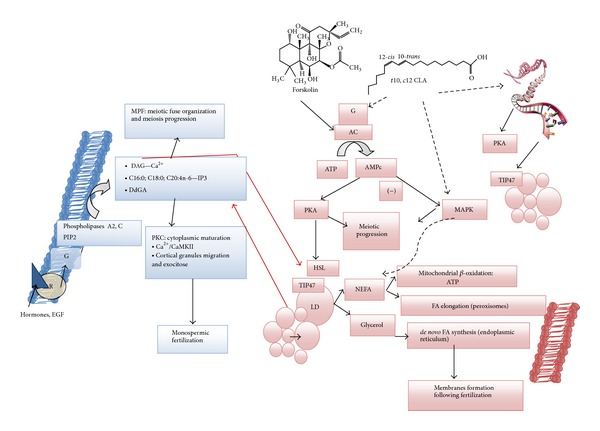
Hypothetical model for the effects of forskolin and* trans*-10,* cis*-12 conjugated linoleic acid (CLA) in the regulation of oocyte lipid metabolism and developmental competence acquisition: red colour and solid arrows, stimulation of cAMP intracellular levels, PKA and MAPK pathways by forskolin (see text for details); potential mechanisms of CLA action are also in red but illustrated by dashed arrows, including stimulation of PKA and MAPK pathways interfering with LD lipolysis, control of oocyte gene expression, and protein synthesis, namely, perilipins. Left, the cytoplasmic membrane and different events that are crucial for suitable oocyte maturation are represented in blue. R and G are the transmembrane G-protein-coupled receptor activated by different hormones or EGF (blue triangle) during this process. Red arrows illustrate the activity of different intracellular messengers and fatty acids regulating oocyte lipid metabolism and quality. DAG, diacylglycerol, DdGA, diradilglyceride, PIP2, phosphatidylinositol 4,5 bisphosphate, IP3, inositol 1,4,5-trisphosphate; PKA, protein kinase A; PKC, protein kinase C, MAPK, mitogen protein kinase; AC, adenylyl cyclase; HSL, hormone sensitive lipase; MGL, monoglyceride lipase; P, perilipin protein; LD, lipid droplet; NEFA, nonesterified fatty acids.

Lipid modulators are substances that are capable of reducing and/or modifying intracellular lipid content of cells. These substances have been successfully applied in ART, namely, in oocyte maturation and embryo production [10, 63, 64]. The* trans*-10,* cis*-12 conjugated linoleic acid (*t*10,* c*12 CLA) is one of these substances, being capable of interfering with lipid accumulation and metabolism in pig adipose explants as well as in pig and cattle oocytes and embryos [9, 11, 53, 65]. In fact, when pig and cattle COC were matured with* t*10,* c*12 CLA, this isomer was accumulated in both, oocyte and CC, changing their FA profiles, especially that of CC [9, 11]. Moreover, the presence of* t*10,* c*12 CLA interfered with oocyte colour tone and probably in LD movements and aggregation during maturation [10, 45, 66]. The exact mechanism through which this isomer influences oocyte lipid metabolism remains elusive. Nevertheless, the* t*10,* c*12 CLA appears to affect the PKA signal transduction pathway, and thus the cAMP cascade of reactions [67] (Figure 4). On the other hand, an increase in lipolysis and in cytosolic perilipin associated with smaller LD was identified in human adipocytes cultured in the presence of this CLA isomer [68]. Therefore, it is possible that PKA and MAPK/ERK pathways may be regulated by* t*10,* c*12 CLA, thus interfering with LD lipolysis (Figure 4) and FA content of oocytes. The analysis of FA and DMA composition of pig COC showed that independently from cell type, CLA treatment reduced the proportions of several individual FA and plasmalogens DMA-16:0,* c*9-16:1, 18:3 n-6, and tended to reduce* c*7-16:1,* c*11-18:1, and 20:4 n-6 [11]. The released FA or the accumulated* t*10,* c*12 CLA may follow the mitochondrial *β*-oxidation to produce energy for maturation progression or FA synthesis to be used in cell formation during embryo development [7, 46].

The diterpenoid forskolin is a chemical stimulator of lipolysis through the activation of adenyl cyclase, whose effects in pig have also been demonstrated in both oocytes and embryos [69, 70]. In a recent experiment, forskolin exposure in different incubation times (44 h, 22 h and 2 h) interfered in oocyte lipid content and LD morphology and impaired fertilization beyond 2 h of supplementation, since it delays meiotic progression and oocyte growth [10]. This supplementation of pig COC culture medium with forskolin, during the initial 2 h of IVM, influenced both oocyte and their CC FA and plasmalogens composition, although their total contents were not affected [11]. Depending on dose and exposure time, forskolin treatment may induce a higher modification in intracellular lipids [10, 11, 70]. As referred, the cytoplasmic maturation in the developing oocyte implies LD movements that can induce LD coalescence and thus morphology modifications. Moreover, by stimulating lipolysis, intracellular LD content may also be modified due to shrinkage [46]. Once lipolytic substances bind to the catalytic subunit of the adenylyl cyclase enzyme (Figure 4), the cAMP is synthesized from the available ATP in the cytoplasm. Consequently, the increased level of cAMP is responsible for the activation of PKA [13, 34]. In turn, PKA phosphorylates endogenous lipases, as the hormone-sensitive lipase (HSL) and also perilipin protein located at the LD surface [13]. Following phosphorilation, HSL is translocated to the cytoplasm where it binds to LD surface protein to induce fragmentation of large droplets into smaller ones, thus increasing accessible droplet surface and the degradation of its core [13, 46]. In the lipolysis of intracellular lipids, it is considered that HSL catalyses triglycerides and diglycerides, while monoglyceride lipase is required to obtain complete hydrolysis of monoglycerides [13, 45, 46, 69]. Therefore, sterol metabolism might be affected by the utilization of lipolytic agents such as adenylyl cyclase stimulators, during oocyte maturation progression, interfering with the acyltransferase activity in translocating the release of cholesterol to the mitochondria, to be metabolised in pregnenolone and progesterone [8]. Moreover, the increase in available glycerol and free FA may induce* de novo* synthesis of long chain FA by reesterification and phospholipids formation for membrane assembly during embryo development [45, 69]. Simultaneously, free FA may be used to produce energy through the *β*-oxidation pathway, to boost maturation progression as in cattle and pig oocytes [7].

Alternatively, PES is another lipid modulator that regulates embryo metabolic pathways. PES increases glucose metabolism through PPP during embryo culture [63]. Hence, PES is a strong electron acceptor that readily oxidizes NADPH to NADP+, thus decreasing NADPH required for the synthesis of numerous lipids, particularly long-chain FA, and reducing intracellular lipid content of embryos [5, 63, 64]. While the major intent of reducing intracellular lipid content is the improvement of blastocyst cryoresistance for subsequent embryo transfer, more research is needed to choose the best pharmacological tools to enhance ART results.

### 4.2. Oocytes and Embryos Cryopreservation: Effects of Lipid Content Modulation/Reduction

Considerable progress has been made in improving and simplifying oocyte and embryo cryopreservation procedures to be routinely used in transfer programs. In general, cryopreservation by slow freezing is a process where extracellular water crystallizes, resulting in an osmotic gradient that draws water from the intracellular compartment until intracellular vitrification occurs [6, 71]. On the other hand, in cryopreservation through vitrification, both intra- and extracellular compartments vitrify after cellular dehydration has already occurred [71]. These cryopreservation techniques have been improved to minimise damage and help oocytes and embryos of different developmental stages to regenerate through several strategies, using microsurgical manipulation, cytoskeletal relaxants (such as cytochalasin B or D), membrane and protein stabilisers, centrifugation, adjusting the concentration of cryoprotectants and/or reducing the cooling volume to a minimum [6, 70, 72–74]. Nevertheless, the success rate is still limited particularly in oocytes.

The plasma membrane of oocytes and embryos is the first cellular structure whose integrity is affected by thermotropic phase transition. During cooling, irreversible damage occurs shortly after exposure to low, but not freezing, temperatures just below 15°C [75, 76]. In oocytes, the greater cellular volume and higher cytoplasmic lipid content increase chilling sensitivity when compared to embryo cells [77]. Furthermore, the less submembranous actin microtubules present in oocytes account for a less robust membrane and, thus, cryopreservation can cause disorganization of cytoskeleton and meiotic spindle, as well as chromosome and DNA abnormalities [37, 77]. Changes in cellular chemical composition and LD association to other organelles or to the cytoskeleton were also identified in GV oocytes and phase separations in the cytoplasm and/or internal membranes in embryos [37, 75, 76].

Differences in LD colour tone of fresh immature and vitrified-warmed pig oocytes were identified: gray in fresh and slightly dark in vitrified oocytes [37]. However, LD size or distribution was similar. On the contrary, Isachenko et al. [78] showed that the two types of LD found in pig oocytes, dark and “gray,” changed their morphology during cooling into a spherical form with lucent streaks impairing oocyte developmental competence.

As referred, the high lipid content that has been related to an increased sensitivity to chilling injury during cryopreservation is particularly important in pigs, but also in cattle [6, 37, 70, 79]. Changing the lipid content of pig or cattle embryos by removing LD may have a direct effect on embryo survival during chilling [5, 75, 80]. This process can be performed by mechanical delipidation through polarization of the cytoplasmic LD and subsequent physical removal of excess lipid, increasing the survival rates of cryopreserved embryos [6, 72, 74]. As an alternative to such invasive techniques that can damage the cellular structure, it is possible to improve the success of cryopreservation of* in vitro* produced embryos by eliminating serum from the culture medium, or by inducing chemical delipidation through metabolic manipulation [5, 79]. It has in fact been demonstrated that adding* t*10,* c*12 CLA to serum-containing media during* in vitro* culture of cattle embryos reduced lipid accumulation and significantly improved blastocyst survival following cryopreservation [53, 81]. In oocytes,* t*10,* c*12 CLA was shown to interfere with lipid metabolism, both in cattle and pig, reducing lipid content in pig [9, 10]. Furthermore, by reducing the lipid content of pig oocytes and embryos, forskolin was shown to increase cryosurvival following vitrification [69, 70]. Similarly, PES can be used to reduce cytoplasmic lipid content improving cryosurvival of cattle embryos [82]. However, the use of PES during* in vitro* culture had a limited effect on pig blastocyst survival after vitrification. Nevertheless, PES increased the proportion of morula and blastocyst formation, reducing the index of DNA fragmentation and the cytoplasmic lipid content of cultured blastocysts [83]. Further studies are needed to broaden the use of lipid modulators to improve cryopreservation survival.

## 5. Conclusion

The lipid content of the pig oocyte, as well as the asynchrony between nuclear and cytoplasmic maturation, renders it a good model in the field of oocyte biology research. Knowledge of the pathways and key molecules regulating these processes may highlight therapeutic possibilities to prevent the excessive accumulation or to modulate lipid composition of cytoplasmic droplets. Furthermore, lipid modulators may also be applied in germ cells and embryo cryopreservation to improve livestock production. Finally these molecules might provide tools to overcome lipid metabolic disorders related to infertility.
